# Supplementary material for: A Critical View of the Application of the APEX Software (Aqueous Photochemistry of Environmentally-Occurring Xenobiotics) to Predict Photoreaction Kinetics in Surface Freshwaters
Source: Molecules. 2019 Dec 18;25(1):9. doi: 10.3390/molecules25010009 (PMC7017383; doi:10.3390/molecules25010009)
Supplement: Supplementary file 1 [file molecules-25-00009-s001.zip › 3.2.4_gcc-4.4.0.zip!/3.2.4_gcc-4.4.0/3.2.4_gcc-4.4.0/doc/octave/html/interpreter/Plotting-on-top-of-Images.html]

Plotting on top of Images - Untitled


Next: Color Conversion,
Previous: Representing Images,
Up: Image Processing


---

### 31.4 Plotting on top of Images

If gnuplot is being used to display images it is possible to plot on
top of images. Since an image is a matrix it is indexed by row and
column values. The plotting system is, however, based on the
traditional (x, y) system. To minimize the difference between
the two systems Octave places the origin of the coordinate system in
the point corresponding to the pixel at (1, 1). So, to plot
points given by row and column values on top of an image, one should
simply call `plot` with the column values as the first argument
and the row values as the second. As an example the following code
generates an image with random intensities between 0 and 1, and shows
the image with red circles over pixels with an intensity above
0.99.

```
     I = rand (100, 100);
     [row, col] = find (I > 0.99);
     hold ("on");
     imshow (I);
     plot (col, row, "ro");
     hold ("off");
```
